# Fertility-sparing treatment for endometrial cancer and atypical endometrial hyperplasia in patients with Lynch Syndrome: Molecular diagnosis after immunohistochemistry of MMR proteins

**DOI:** 10.3389/fmed.2022.948509

**Published:** 2022-08-25

**Authors:** Ursula Catena, Luigi Della Corte, Antonio Raffone, Antonio Travaglino, Emanuela Lucci Cordisco, Elena Teodorico, Valeria Masciullo, Giuseppe Bifulco, Attilio Di Spiezio Sardo, Giovanni Scambia, Francesco Fanfani

**Affiliations:** ^1^Division of Gynecologic Surgery, Department of Woman, Child and Public Health, Fondazione Policlinico Universitario Agostino Gemelli, Istituto di Ricovero e Cura a Carattere Scientifico, Rome, Italy; ^2^Department of Neuroscience, Reproductive Sciences and Dentistry, School of Medicine, University of Naples Federico II, Naples, Italy; ^3^Pathology Unit, Department of Advanced Biomedical Sciences, School of Medicine, University of Naples Federico II, Naples, Italy; ^4^Medical Genetics Unit, epartment of Laboratory and Infectious Sciences, Fondazione Policlinico Universitario A. Gemelli IRCCS, Rome, Italy; ^5^Department of Public Health, University of Naples Federico II, Naples, Italy; ^6^Catholic University of Sacred Heart, Rome, Italy

**Keywords:** endometrial cancer, Lynch Syndrome, fertility-sparing treatment, immunohistochemistry, mismatch repair, genetic testing

## Abstract

**Introduction:**

Lynch Syndrome (LS) represents the hereditary condition that is most frequently associated with endometrial cancer (EC). The aim of this study is to assess the presence of Lynch Syndrome (LS) in young women with mismatch repair (MMR)-deficient atypical endometrial hyperplasia (AEH) and non-myoinvasive FIGO G1 endometrioid EC and its possible impact on the outcome of conservative treatment.

**Methods:**

Six MMR-deficient cases identified from a previous cohort of 69 conservatively treated patients were selected to be screened for germline mutations in MMR genes. In each patient, the outcomes of conservative treatment for AEH and EEC, including response, relapse, progression, and pregnancy, were assessed.

**Results:**

Five out of 6 patients underwent genetic test for LS. Three out of these 5 patients showed a positive genetic test. Patient 1 showed the c.942 + 2 T>A heterozygous variant of *MSH2* mutation; after 12 months of complete response, she had relapse and progression of disease. Patient 4 showed the c.2459-1G>C variant of *MSH2* mutation; after complete response, she failed to achieve pregnancy; she had relapse after 24 months and underwent hysterectomy. Patient 6 showed the c.803 + 1 heterozygous variant of *PMS2* mutation; she had relapse of disease after 18 months from the first complete response and then underwent hysterectomy.

**Conclusions:**

In this series, 3 out of 6 women with MMR-deficiency had LS. None of the patients achieved pregnancy, and those who responded to treatment had subsequent relapse of disease. Patients undergoing fertility-sparing treatment for atypical endometrial hyperplasia and endometrial cancer should perform MMR immunohistochemical analysis in order to screen LS.

## Introduction

Endometrial cancer (EC) is the fifth cause of cancer in women and the most common gynecological cancer in developed countries (1–3). Nearly 90% of cases of EC occur in women >50 years of age, with a mean age at diagnosis of 63 years, while 4% occurs in young women under 40 years old (4). Ninety-five percent of EC are sporadic, while 5% are hereditary. The hereditary condition that is most frequently associated with EC is Lynch Syndrome (LS), an autosomal dominant disorder characterized by a germline pathogenic variant of one of the Mismatch Repair (MMR) genes (MLH1, PMS2, MSH2, MSH6) (5), resulting in microsatellite instability (MSI). MSI is a condition in which there is an altered number of repeats of short DNA sequences, called microsatellites, between tumor and normal tissue. MSI might represent the consequence of phenotypic evidence of MMR deficiency (6, 7). MSI analysis and MMR protein immunohistochemistry (IHC) have an important role in diagnosis: two studies reported high concordance between MSI and IHC analysis both in colorectal cancer (CRC) and EC, with discordance in the rare MMR-proficient/MSI-high cases (<1%) in EC patients, probably due to POLE-EDM variants (6, 7), while Ryan et al. reported how IHC outperforms MSI for tumor triage and is a reliable method for identifying both germline and somatic MMR mutations in women with EC (8). Women with LS have an increased risk to develop EC (up to 61% that is 39 times higher than the general population) (9–12) as well as other cancers, including colorectal (up to 57%), ovary (up to 40%), kidney, small bowel and biliary tract cancers (LScarisk.org). The prevalence of LS among ECs ranges from 0.5 to 4.6% (13), although it is probably underestimated. EC could represent a sentinel event of LS, since it is often the first cancer to occur, in over 50% of cases (14, 15). The median age at diagnosis of EC for women with LS is generally lower than in sporadic cases (49 vs. 60 years, respectively) (15). EC is associated with MMR abnormalities and LS has worse prognostic factors and outcomes (10). According to Lu et al., patients with EC and LS tend to have a lower BMI (16), except for patients with MSH6 mutation who seem to have a clinical profile more similar to those with sporadic cancers. However, data on the clinicopathological characteristics of LS-related EC are missing, as the studies are mostly conflicting. In these patients, the tumor is more often in the uterine isthmus and mostly presents as a well-differentiated endometrioid adenocarcinoma (5). However, other studies showed a higher percentage of non-endometrioid histotypes, a higher FIGO stage at presentation, a higher number of G3 tumors, a deeper myometrial invasion, and a higher mitotic index in the LS-related EC (17). The 4 genes responsible for LS have different penetrance and expressivity. The risk of endometrial cancer (EC) is higher especially among carriers of *MSH2* (49%), *MSH6* (41%) and *MLH1* (37%) mutations. Cancer is also common in elderly women with PMS2 mutations (13%) (18, 19). The screening for LS is often based on clinical criteria, such as the Amsterdam criteria and the Bethesda criteria although the latter are of less importance nowadays, which consider age (<50 years), family history of colorectal cancer, positive personal history for cancer of the LS spectrum. The clinical suspect must be confirmed by molecular analysis, which allows the characterization of the patient's genotype. However, the clinical criteria do not always allow to effectively identify the pathogenic variants for LS (20–23). The benefit for universal screening in CRC and EC is well known: Kunnackal John et al. showed how LS screening in EC yielded significantly higher somatic mutations compared to CRC [pooled percentage 16.94 vs. 5.23%, 95% CI 4.93–5.47%—Mann Whitney test, *p* < 0.0001], suggesting the possibility for IHC and somatic mutation testing before germline testing in EC due to higher prevalence of somatic mutations as well as germline testing in these patients and in other major Lynch-associated tumors (24). The early identification of patients with LS is necessary to allow a close follow-up and personalized/conservative therapy, for the patient him/herself and his/her affected relatives.The objective of our study was to assess the presence of LS in young women undergoing conservative management for atypical endometrial hyperplasia (AEH) and non-myoinvasive FIGO IA endometrioid endometrial cancer (EEC), evaluating its possible impact on the response rate, relapse rate, progression rate, and pregnancy rate.

## Materials and methods

The MMR-deficient patients were identified from a previous retrospective cohort of 69 patients submitted to fertility-sparing treatment for FIGO IA G1 EC and AEH, between January 2004 and December 2018. The study was carried out at the “Fondazione Policlinico Universitario A. Gemelli—IRCCS” of Rome and at the University “Federico II” of Naples. All medical records of patients with AEH and EEC belonging to the two centers were retrospectively analyzed.

### Therapy outcome and follow-up

All patients underwent hysteroscopic resection of the pathology followed by progestin therapy: patients 1 and 6 with Megestrol Acetate (Megace) 160 mg daily given orally: patients 2, 3, 4, and 5 with Levonorgestrel releasing Intra Uterine Device (IUD) (Mirena). Oncological outcomes at histologic examinations were defined as complete response (CR), stable disease (SD), progression (P) and relapse (R). CR was defined as the complete disappearance of AEH or EEC; SD as persistence of AEH or EEC; P as progression of AEH to EEC or worsening of the histological grade of EEC. R was defined as the presence of EEC or AEH after CR had been previously achieved. In agreement with international guidelines (25, 26), the presence of at least two consecutive CR was defined as “regression of disease”, while the lack of two consecutive CR was labeled “resistance”. The reproductive outcome was assessed as the achievement of a successful pregnancy. For every patient, we collected pathology reports of hysteroscopic biopsies at 3, 6, 12 and up to 27 months after treatment, as well as data on pregnancies (spontaneous delivery, cesarean section, miscarriages).

### Screening and sequencing procedure

Cases were labeled as “MMR-deficient” based on immunohistochemical screening for MLH1, MSH2, MSH6, and PMS2 proteins (25). In patients with lack of MLH1 expression, MLH1 promoter methylation was analyzed by MS-MLPA (Mrc Holland) in order to exclude somatic hypermethylation. In patients with MMR-d tumors, screening for germline mutations in MMR genes was conducted by Next Generation Sequencing on Ion Torrent PGM with a homemade 4-genes panel (*MSH2, MSH6, MLH1, PMS2*). Sequencing data of the targeted genes were analyzed with Torrent Suite (Life Technologies). Additional Sanger sequencing was performed for regions containing putative variants. Exon deletions and duplications were assessed by MPLA (Mrc Holland). Alterations were classified based on guidelines established by Insight (2018-06_InSiGHT_VIC_v2.4) into the following categories: (5) pathogenic variant (PV); (4) variant, likely pathogenic; (3) variant, unknown significance; (2) variant, likely benign; (1) benign.

### Ethical statement

The study received approval from the Institutional Review Board of the Catholic University of the Sacred Heart of Rome and the University of Naples Federico II (Prot. No. 0048361/20). All included patients signed informed written consent for the use of their biospecimens for research purposes and all data were anonymized in order to avoid the identification of the subjects. The whole study was performed following the Declaration of Helsinki.

## Results

From the case history of our previous study, including 69 patients [47 (68.1%) with AEH and 22 (31.9%) with EEC] (25), six (8.7%) (3 with AEH and 3 with EEC) were enrolled in our study based on a deficient pattern of expression of the MMR proteins. The patient's characteristics are resumed in Table 1. Of the 6 MMR-deficient patients, one had MSH2/MSH6 deficiency (EEC), 3 had MSH6 deficiency (1 EEC and 2 AEH) and 2 had PMS2 deficiency (1 EEC and 1 AEH) (Table 2). Five out of 6 patients received the diagnosis of EEC/AEH before 40 years old, with mean age at diagnosis of 36 (± 4.28 SD) years old (range 31–43). The mean BMI was 24.9 (± 7.26 SD) with only one patient with BMI higher than 30 kg/m^2^ (39.3 kg/m^2^) (Table 1). None of the patients reported tumor risk factors for EC (diabetes, hypertension, PCOS); 2 (33.3%) had a positive family history for neoplasms of the LS spectrum (patient 1 for CRC, patient 4 for EC). The dosage of CA125 was negative (<35 UI/mL) in all patients.

**Table 1:** Patients' characteristics.

**Patients**	**Age at** **diagnosis** **(years)**	**BMI** **(kg/m^2^)**	**Tumor** **risk factors**	**Previous** **pregnancy**	**Symtomps**	**Family history for** **endometrial** **cancer**	**Family history for** **other SL-related** **cancers**
1	33	19.5	No	No	None	No	Yes (Colorectal)
2	43	21.36	No	No	Metrorrhagia	No	No
3	31	39.3	No	No	Metrorrhagia	No	No
4	38	22.4	No	No	None	Yes	Yes (Colorectal)
5	37	24.61	No	No	Metrorrhagia	No	No
6	34	22	No	Yes^*^	Metrorrhagia	No	No

BMI, body mass index;

* 2 previous vaginal delivery.

**Table 2:** Diagnosis, MMR-deficiency at immunochemistry, genetic test result with variant identified in patients 1, 4, and 6 and located in a recognized site of splicing (±1 o ±2), and outcomes of the 6 patients (EEC, Stage IA G1 endometrioid endometrial cancer; AEH, atypical endometrial cancer; PR, partial response; CR, complete response; SD, stable disease; R, relapse; P, progression; ^§^THL + SOB; ^*^for patient's choice; ^**^drop-out; ^a^LNG-IUD from the 6th month).

**Patients**	**Diagnosis**	**MMR-d**	**Mutated** **gene**	**Nucleotide** **or** **amino acid** **substitution^@^**	**IARC** **Classification**	**Medical** **therapy**	**3** **months** **follow-up**	**6** **months** **follow-up**	**12** **months** **follow-up**	**15** **months** **follow-up**	**18** **months** **follow-up**	**21** **months** **follow-up**	**24** **months** **follow-up**	**27** **months** **follow-up**	**42** **months** **follow-up**	**Pregnancy**	**Radical** **surgery^§^**
1	Stage IA G1 EEC	MSH6/ MSH2	*MSH2*	c.942+2T>A p.(Val265_ Gln314del)	Class 4 (likely pathogenetic)	Megace^®^	SD (AEH)	SD^a^	CR	CR	CR	CR	R; P	/	R	No	Yes
2	Stage IA G1 EEC	MSH6	/	/	/	Mirena^®^	SD	SD	SD	/	/	/	/	/	/	No	Yes
3	Stage IA G1 EEC	PMS2	/^**^	/	/	Mirena^®^	SD	SD	CR	R	/	/	/	/	/	No	Yes
4	AEH	MSH6	*MSH2*	c.2459-1G>C p.(Gly820A lafs^*^3)	Class 4 (likely pathogenetic)	Mirena^®^	CR	CR	CR	/	/	/	/	R	/	No	Yes^*^
5	AEH	MSH6	/	/	/	Mirena^®^	CR	CR	CR	/	/	/	/	/	R	No	Yes
6	AEH	PMS2	*PMS2*	c.803+1G>T p.(Leu236His fsTer30)	Class 4 (likely pathogenetic)	Megace^®^	CR	CR^a^	CR	CR	CR	R	SD	/	/	No	Yes

@ The methods analysis included: Next Generation Sequencing on Ion Torrent PGM for the screening of germline mutations in MMR genes, Torrent Suite (Life Technologies) for the sequencing of the targeted genes, Sanger sequencing for the sequencing of regions containing putative variants, and MPLA (Mrc Holland) for the assessment of exon deletions and duplications.

### Results of genetic tests

The genetic testing was carried out on 5 of the 6 MMR-d patients because one patient refused. In patient 1 with MSH2 deficiency on tissue sample, the c.942 + 2 T>A heterozygous variant has been identified by Next Generation Sequencing (NGS). This sequence change affects a donor splice site in intron 5 of the *MSH2* gene. It is expected to disrupt RNA splicing and likely to cause the skipping of exon 5, resulting in an abnormal protein, p.(Val265_Gln314del), or a transcript that is subject to nonsense-mediated mRNA decay. This variant has been classified as Likely Pathogenic (class 4 IARC) (27) according to the Insight criteria (Table 2). In patient 2, who showed MSH6 deficiency on tissue sample, genetic testing did not reveal any mutation. Patient 3, with PMS2 deficiency on tissue sample, refused consent for the genetic test. In patient 4, exhibiting MSH6 deficiency on tissue sample, the c.2459-1G>C heterozygous variant has been identified by NGS. This sequence change affects an acceptor splice site in intron 14 of the *MSH2* gene. It is expected to disrupt RNA splicing and likely to cause skipping of exon 15, resulting in an absent or disrupted protein product leading to the formation of a premature stop codon after 3 amino acids p.(Gly820Alafs^*^3). This variant has been classified as Likely Pathogenic (class 4 IARC) (27) according to the Insight criteria (Table 2). In patient 5 with MSH6 deficiency on tissue sample, the genetic test did not reveal any mutation. In patient 6, who showed PMS2 deficiency on tissue sample, the c.803 + 1 heterozygous variant of the coding sequence of the PMS2 gene has been identified. This sequence change affects a donor splice site in intron 7 of the *PMS2* gene. It is expected to disrupt RNA splicing and likely to cause the skipping of exon 7 leading to the formation of a premature stop codon after 30 amino acids p(Leu236HisfsTer30). This variant has been classified as Likely Pathogenic (class 4 IARC) (27) according to the Insight criteria (Table 2).

### Oncological and reproductive outcomes

In patient 1 SD with AEH on the first follow-up and CR at 12 months was observed. After further 12 months (at 24 months follow-up), she had R with P to EEC. Despite adequate counseling where the need for radical surgery was explained, she chose to maintain medical therapy with a close follow-up every 3 months, given her strong desire for offspring. After further 18 months (at 42 months follow-up), she had a new R to EEC and she finally decided to undergo hysterectomy. Patient 2 decided to undergo hysterectomy after stable disease SD at 3, 6 and 12 months of follow-up. Patient 3 showed CR at 12 months; however, CR was not confirmed in the subsequent follow-up biopsy (at 15 months follow-up), and the patient chose to undergo hysterectomy. Patient 4 had CR at 3, 6 and 12 months. After unsuccessful attempts to get pregnant, she showed R after 24 months from the initial CR (at 27 months follow-up). Thus, she decided to undergo hysterectomy. Patients 5 had CR on 4 consecutive biopsies. After unsuccessful attempts, she had R after 39 months of initial CR (42 months follow-up) and underwent hysterectomy. Patient 6 had CR after 3 months but developed R 18 months later (at 21 months of treatment) with SD on the subsequent biopsy, and underwent hysterectomy (Table 2). In all patients that underwent hysterectomy, the pathology report of the surgical specimen confirmed the hysteroscopic diagnosis. No patients showed a recurrence with a median follow-up of 20 months.All patients tried to get pregnant spontaneously and during all follow-up period, without resorting to any medical therapy.MMR-deficient (dMMR) cases, defined by lack of MMR protein expression detected by IHC analysis of tumor tissue, showed resistance to treatment more commonly than MMR-proficient (pMMR) cases [2 (33.3%) vs. 10 (15.9%)], with a RR of 2.1 (95%CI: 0.6–7.5) but with no statistical significance (*p* = 0.2508). Recurrence of AEH/EEC after a complete regression occurred significantly more commonly in dMMR cases than pMMR cases [6 (100%) vs 17 (26.4%)], with a RR of 3.8 (95%CI: 2.4–5.9, *p* < 0.0001). In predicting recurrence of disease after a complete regression, a deficient immunohistochemical expression of MMR showed sensitivity = 22.2%, specificity = 100%, and AUC = 0.61 (95%CI: 0.44–0.76) (21).

## Discussion

In this study, we considered 6 MMR-deficient cases of conservatively treated AEH and EEC from a cohort of 69 patients (25). The conservative managemet included hysteroscopic resection followed by local or systemic drug theraphy: 36 (52.2%) women underwent LNG-IUD insertion, and 33 (47.8%) MA administration. Overall, 17.4% of women showed resistance to treatment, while 31.6% of women who responded showed a subsequent recurrence. Out of 5 patients who underwent genetic test, 3 (60%) were carriers of a germline variant of MMR genes: 2 patients showed a pathogenic mutation of *MSH2* and 1 patient of *PMS2*. All patients with confirmed LS responded to conservative treatment; however, all failed to achieve pregnancy and had relapse of disease. The rate of LS endometrial cancer patients in this series (4.3%) was almost superimposable to that reported in the literature (11). Among MMR-deficient cases, recurrence occurred after 24 and 39 months in the LNG-IUD group, and after 12 and 18 months in the MA group. In predicting recurrence of disease after a complete regression, a deficient immunohistochemical expression of MMR showed sensitivity = 50%, specificity = 100%, and area under the curve (AUC) = 0.75 (95%CI: 0.00–1.00) in the LNG-IUD subgroup, and sensitivity = 14.3%, specificity = 100%, and AUC = 0.57 (95%CI: 0.35–0.79) in the MA subgroup (25). The lifetime risk of developing cancer is significantly higher in patients with MSH2, and MLH1 mutations compared to PMS2 and MSH6 mutations. Patients with MLH1, MSH2, and MSH6 mutations have a rapidly rising risk of gynecological cancers from 40 years of age (18). In these patients, the incidence of EC is 51% among carriers of MSH2 mutation, 49% among carriers of MSH6 mutation, 34% among carriers of MLH1 mutation. In our study, 2 patients (MSH2 mutation confirmed) had a family history for colorectal cancer and both colorectal cancer and EC, respectively. In 2016, Rubio et al. reported how all patients with pathogenic mutations in any of the MMR genes had a family history (first-degree relatives) compatible with LS, but more than half (61.79%) of patients with no pathogenic mutation had a positive family history. These differences are explained by a selection bias related to one of the inclusion criteria concerned the family history of cancer of the LS spectrum (20). In 2019, a review and meta-analysis estimated that only 56% of cases of LS are diagnosed based on traditional clinical-anamnestic indicators, while 43% of cases would be lost and undiagnosed if we exclusively used these criteria. This provides further support to the current data present in literature, suggesting the need for a universal screening approach to all new cases of EC arising in young women, in order to maximize the detection of LS patients (28). In our study, we used IHC analysis as a screening method, but many studies also propose the analysis of microsatellites. In 2016, Rubio et al. used both methods, demonstrating high sensitivity and specificity in selecting patients with LS mutations (20). In particular, a study by Leenen et al. (29) on 183 women showed a 100% agreement between the two techniques and Walsh et al. (30) reported similar results (97.5%). It is estimated that the specificity of the MSI analysis for LS is around 90.2% and that the sensitivity is 91% for MLH1/MSH2 and 77% for MSH6/PMS2. The specificity and sensitivity of the IHC analysis, on the other hand, are respectively 88.8 and 83% (31). A universal screening approach would certainly have considerable economic implications; for this reason, it is essential to aspiring to cost optimization. The analysis of MSI has limitations, represented by the inability to discriminate the type of protein of the MMR deficient and also many MMR-d tumors for MSH6 are low instability (MSI-L), therefore, this technique cannot be very sensitive in detecting many low-penetrance MSH6 germline mutations. The IHC analysis has lower costs. Thus, the best screening approach, in terms of cost-effectiveness, would therefore be to start with an IHC investigation of the expression of MMR proteins in order to limit further costs and to refer patients with IHC MMR-d phenotype to genetic testing at a later time or, in case of strong clinical suspicion, despite the expression of MMR proteins being intact (19). Furthermore, in the event that the IHC shows a deficit of expression of MLH1 or PMS2, it is advisable to first perform the analysis of methylation of the MLH1 promoter, which in most cases allows excluding sporadic forms of EC from MSI, although this methylation is rarely the consequence of a germline mutation of the promoter. Since a germline mutation of the MLH1 promoter is configured as a rare event, in the absence of a personal or family history strongly suggestive of a hereditary process, for which a genetic analysis would be carried out on the promoter, the methylation of the promoter is considered a fairly reliable indicator of sporadic cancer (19). Considering that IHC is a highly sensitive technique for identifying mutations in MMR genes in CRC, it could be expected that an IHC-based screening approach could prevent a significant number of LS patients remain undiagnosed (19). Our sample was analyzed for the IHC expression of MMR proteins using two different criteria in the two centers. Patients from Fondazione Policlinico A. Gemelli—IRCCS of Rome were analyzed for all four proteins of the MMR (MSH2, MSH6, PMS2, MLH1); those of the University of Naples center were analyzed exclusively for two proteins, MSH6 and PMS2. The latter approach agrees with two studies (32, 33), according to which an initial screening limited to two proteins can significantly reduce costs without affecting efficacy—as MSH6 and PMS2 are mandatory partners, respectively, of MSH2 and MLH1—whereby the lack of expression of these proteins reflects a deficit of their partners. The evaluation of the clinical and anamnestic data of our patients with pathogenic variants has also revealed a tendentially lower BMI, compared to women with sporadic forms of EC, and negativity for the normal risk factors typical of sporadic forms of EC, in accordance with data in literature. It is important to identify LS patients among new EC because these patients have an increased risk to develop other types of cancers of the LS spectrum, synchronous or metachronous, allowing such patients to benefit from close surveillance (through colonoscopy and transvaginal ultrasound) and possible preventive interventions (34). Furthermore, MMR status is starting to acquire prognostic value, as studies are beginning to demonstrate differences in characteristics and outcomes between MMR-proficient and MMR-deficient EC (35). In presence of young women with Stage IA G1 EEC or AEH, strongly persuaded to preserve their fertility and candidates for conservative treatment (36–38), identifying MMR-deficient patients and, possibly, patients with LS could also be essential in guiding adequate counseling: indeed, our data show how these patients tend to have a worse outcome than MMR-proficient patients (39, 40). In detail, all patients with confirmed LS responded to conservative treatment in our series. However, none achieved pregnancy, and all had relapse of disease. This underlines the unfavorable impact of LS on the outcomes of AEH and EEC. As a consequence, the search for tailored treatment strategies for women affected by LS could provide a good strategy to maximize clinical benefit. Further studies are necessary to assess whether a successful pregnancy may be achieved by lengthening the relapse-free period. The comprehension of predictive genetic testing for LS by patients is fundamental to avoid refusals, as happened in our case with patient 3, also involving families considering that they often play an important role in the decision compared to health professionals; also, the deconstruction of current misconceptions related to potential abuses of genetic information, the emphasis of clinical utility of genetic assessment, and the use of genetics to the specific context of cancer care is crucial for patients' inclusion with newly diagnosed cancer of LS spectrum in clinical cancer genetics services (41). Genetic analysis in women treated conservatively for AEH/EEC under 45 years old can help to find LS families that would not have been identified using existing criteria and to provide them adequate counseling regarding screening of other cancers of the LS spectrum. The screening procedure could begin, as for colorectal cancer, with the IHC analysis, although it has limitations as abovementioned. The mutations found in our sample, although small, are different from those described more frequently for every single gene and therefore there is the need to intensify genetic studies to identify a greater number of pathogenic variants that would allow us to diagnose not only spectrum tumors of LS but also other types of cancer. The need for biomolecular and genetic prognostic factors that can facilitate decision making is nowadays essential (42, 43). On the whole, prospective and larger population studies are needed to evaluate the applicability and usefulness of a “screening test” for LS in young women diagnosed with AEH and EEC which could be based on IHC analysis and then select among these, patients eligible to carry out the genetic test.

## Data availability statement

The datasets presented in this study can be found in online repositories. The names of the repository/repositories and accession number(s) can be found in the article/supplementary material.

## Ethics statement

The study was conducted according to the guidelines of the Declaration of Helsinki and approved by the Institutional Review Board of the Catholic University of the Sacred Heart of Rome and the University of Naples Federico II (Prot. No. 0048361/20). The patients/participants provided their written informed consent to participate in this study. Written informed consent was obtained from the individual(s) for the publication of any potentially identifiable images or data included in this article.

## Author contributions

UC and LDC contributed to conception of the study, interpretation of data, statistical analysis, and drafting the article. AR, AT, and EL contributed to acquisition and interpretation of data and drafting the article. EL contributed to acquisition and interpretation of data. VM, GB, and AD contributed to drafting the article. GS and FF contributed to review the article critically for important intellectual content and final approval of the version to be published.

## Conflict of interest

The authors declare that the research was conducted in the absence of any commercial or financial relationships that could be construed as a potential conflict of interest.

## Publisher's note

All claims expressed in this article are solely those of the authors and do not necessarily represent those of their affiliated organizations, or those of the publisher, the editors and the reviewers. Any product that may be evaluated in this article, or claim that may be made by its manufacturer, is not guaranteed or endorsed by the publisher.
